# The Role of Post-Neoadjuvant Chemotherapy Tumor Volume for Prognostication and Treatment Guidance in Loco-Regionally Advanced Nasopharyngeal Carcinoma

**DOI:** 10.3390/cancers11111632

**Published:** 2019-10-24

**Authors:** Fo-Ping Chen, Dan-Wan Wen, Feng Li, Li Lin, Jia Kou, Wei-Hong Zheng, Li Li, Guan-Qun Zhou, Ying Sun

**Affiliations:** 1 Department of Radiation Oncology, State Key Laboratory of Oncology in South China, Sun Yat-sen University Cancer Center, Guangdong 510060, China; 2 Key Laboratory of Nasopharyngeal Carcinoma Diagnosis and Therapy, Collaborative Innovation Center for Cancer Medicine, No. 651 Dongfeng Eastern Road, Guangdong 510060, China; 3 Department of imaging Diagnosis and Interventional Center, State Key Laboratory of Oncology in Southern China, Sun Yat-sen University Cancer Center, No. 651 Dongfeng Eastern Road, Guangdong 510060, China

**Keywords:** nasopharyngeal carcinoma, intensity-modulated radiation therapy, tumor volume, concurrent chemotherapy, neoadjuvant chemotherapy, treatment outcome

## Abstract

The value of post-neoadjuvant chemotherapy (NACT) tumor volume for prognostication in loco-regionally advanced nasopharyngeal carcinoma (LA-NPC) is unascertained. Here, we recruited 4109 histologically proven LA-NPC (stage III-IVA) that were treated with radical chemo-intensive-modulated radiotherapy (IMRT). Post-NACT gross primary tumor (GTVp) and lymph node (GTVnd) volumes of each patient were calculated from planning computed tomography (CT). We observed similar linear association between GTVp/GTVnd and overall survival (OS); thresholds of 52 cm^3^ for GTVp and 12 cm^3^ for GTVnd were consistent for risk discretization for OS, disease-free survival (DFS), distant metastasis-free survival (DMFS), and local relapse-free survival (LRFS). Recursive partitioning analysis (RPA) modelling incorporating T-/N-categories and GTVp/GTVnd yielded four T-N-volume (TNV) risk groupings with disparate OS (*p* < 0.001). TNV risk stratification outperformed GTVp/GTVnd and eighth edition TNM for predicting OS (AUC 0.643 vs. 0.541–0.591; *p* < 0.001), DFS (0.629 vs. 0.545–0.580; *p* < 0.001), and DMFS (0.652 vs. 0.522–0.621; *p* < 0.001). NACT + concurrent chemoradiotherapy (CCRT) over NACT + IMRT was not superior for low- and low–intermediate-risk groupings (*p* > 0.05 for both), but superior for intermediate- and high-risk groupings in terms of OS (HR 0.68 (95% CI 0.47–0.99) for intermediate risk, 0.73 (0.55–0.97) for high risk; both *p* < 0.05). Overall, GTVp/GTVnd represent effective indicators for prognostication and decision-making in LA-NPC after NACT.

## 1. Introduction

Nasopharyngeal carcinoma (NPC) arises from the nasopharyngeal epithelium and has an extremely unbalanced geographical distribution, with a high incidence in southern China and the surrounding areas [1]. Radiotherapy (RT) is usually the mainstay treatment modality for NPC, due to anatomic constraints and its radiosensitivity [2]. For patients with loco-regionally advanced NPC (LA-NPC), chemo-radiotherapy is essential because of the poor prognosis of these patients [3]. Among the chemotherapy regimens, neoadjuvant chemotherapy (NACT) is increasingly recommended given its advantages of better tolerance and earlier eradication of micrometastases [4,5,6]. Importantly, more than 90% of patients with LA-NPC have shown tumor response (complete response [CR] or partial response [PR]) to NACT and demonstrated favorable outcomes after radical treatment [7]. Nonetheless, the concurrent chemoradiotherapy (CCRT) followed NACT is widely recognized as high toxicity and poor tolerance; only about 30–39% of patients could complete three cycles of concurrent chemotherapy after NACT [4,6]. This raises interesting questions regarding the necessity of concurrent chemotherapy for the patients with LA-NPC but having good response to NACT. However, there is still no efficient tool to distinguish the NACT-sensitive patients who may be potentially safe to omit concurrent chemotherapy after NACT. Therefore, identification of an effective response evaluation index for NPC after NACT to inform on the risk of distant metastasis/loco-regional relapse for treatment intensification or de-intensification is essential.

Response evaluation in solid tumor after treatment is usually performed by measuring the shrinkage or enlargement in maximum diameter [8]. However, it is difficult for NPC to evaluate the response to NACT using conventional response evaluation criteria, as NPC is irregular shaped and usually involves the bone structure of the skull base in locally advanced patients. Thus, the conventional response evaluation method may not be applicable for NPC. Contrarily, the tumor volume may be a more valuable indicator of treatment response than unidimensional measurements, because it provides three-dimensional information, especially the remaining burden of the tumor after NACT [9,10,11]. Hence, post-NACT tumor volume may reflect the chemotherapeutic sensitivity of NACT and can be used for risk stratification for NPC. Nonetheless, previous studies were mainly focused on the prognostic significance of tumor volume prior to treatment [9,10,11,12,13,14], and the potential clinical utility of post-NACT tumor volume for prognostication and decision-making guidance is underdetermined.

According to this premise, we undertook a large-scale analysis in LA-NPC who were treated with NACT plus CCRT/RT alone, to investigate the prognostic value of post-NACT tumor volume on treatment outcomes in the era of intensive-modulated radiotherapy (IMRT). To our knowledge, this is the first and largest experience to assess the value of post-NACT tumor volume on response evaluation in NPC to date.

## 2. Results

### 2.1. Patient Characteristics and Treatment Outcomes

The clinicopathological characteristics of the 4109 patients are summarized in Table 1. Median post-NACT gross tumor (GTVp) and lymph node (GTVnd) volumes were 48.3 cm^3^ (interquartile range (IQR), 28.4–77.5 cm^3^) and 11.2 cm^3^ (4.8–22.1 cm^3^), respectively. Within the median follow-up of 52.8 months (IQR 41.2 to 67.6 months), we recorded 475 (11.6%) loco-regional relapse, 631 (15.4%) distant metastasis, and 645 (15.7%) death. The five-year overall survival (OS), disease-free survival (DFS), distant metastasis-free survival (DMFS), and local relapse-free survival (LRFS) were 84.0%, 74.3%, 83.3%, and 86.6%, respectively.

### 2.2. Prognostic Value of GTVp and GTVnd on Survivals

**Figure 1:**
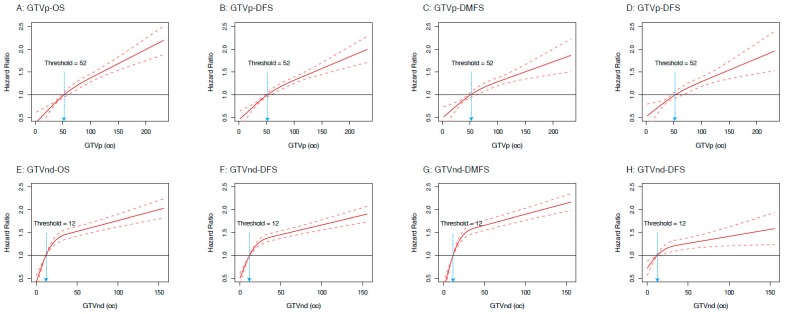
Estimated hazard ratios (HRs) (solid lines) with 95% confidence intervals (dotted line) for the association of post-neoadjuvant chemotherapy gross primary tumor (GTVp) and lymph node (GTVnd) volumes with overall survival (OS), disease-free survival (DFS), distant metastasis-free survival (DMFS), and local relapse-free survival (LRFS) in 4109 patients. The prognostic effects of GTVp on the risks of treatment outcomes are modeled with a restricted cubic splines (RCS) expansion, with GTVp/GTVnd as continuous covariates. The risk (in HR) of OS, DFS, DMFS, and LRFS increased along with the augment of GTVp.

Through restricted cubic splines (RCS) analyses, we observed that the risks (in hazard ratio) of OS, DFS, DMFS, and LRFS increased along with the augment of GTVp and GTVnd (Figure 1). We also identified thresholds of 52 cm^3^ for GTVp and 12 cm^3^ for GTVnd that were consistent for risk discretization to OS, DFS, DMFS, and LRFS. Hence, we transformed the GTVp and GTVnd into categorical variables based on these thresholds. We then investigated the prognostic value of GTVp and GTVnd by using multivariable analyses. We observed that GTVp was independently significant for OS (hazard ratio (HR) 1.63, 95% confidence interval (CI) 1.35–1.97; *p* < 0.001; Supplementary Table S1), DFS (1.45, 1.25–1.68; *p* < 0.001; Supplementary Table S1), and DMFS (1.52, 1.26–1.84; *p* < 0.001; Supplementary Table S1), while GTVnd was independently significant for OS (HR 1.79, 95% CI 1.49–2.14; *p* < 0.001; Supplementary Table S1), DFS (1.70, 1.48–1.96; *p* < 0.001; Supplementary Table S1), DMFS (1.73, 1.44–2.08; *p* < 0.001; Supplementary Table S1), and LRFS (1.61, 1.31–1.97; *p* < 0.001; Supplementary Table S1), after adjusted for age, gender, family history, and treatment scheme.

### 2.3. Construction of TNV Risk Grouping Using Tumor Volume and T-, N-Category

**Figure 2:**
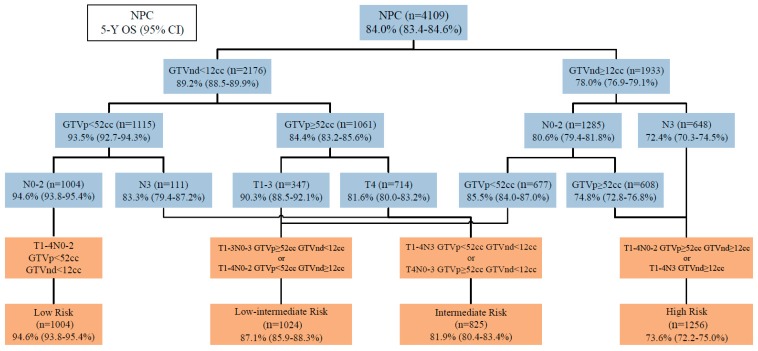
The T-N-volume (TNV) risk stratifications derived by recursive partitioning analysis (RPA).

**Figure 3:**
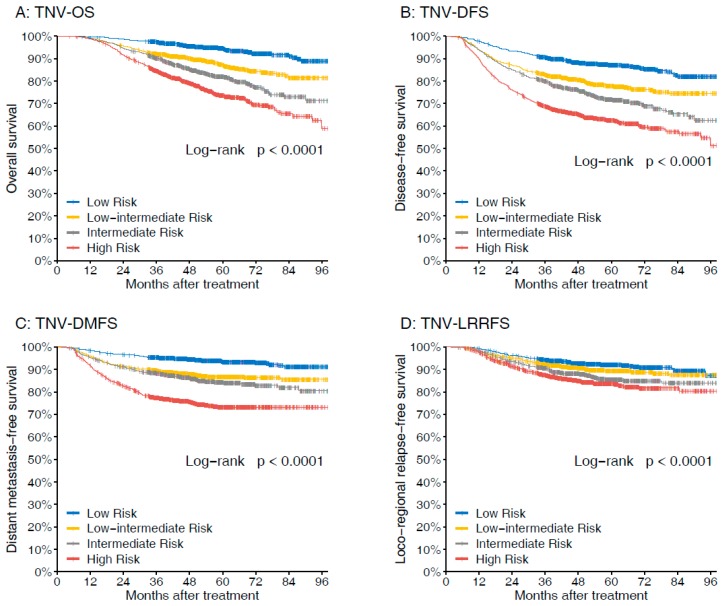
Comparison of the survival rates of different TNV groups with regard to (**A**) overall survival, (**B**) disease-free survival, (**C**) distant metastasis–free survival, and (**D**) loco-regional relapse-free survival.

Considering the prognostic value of the volumes for LA-NPC, we then incorporated GTVp (<52 cm^3^ or ≥52 cm^3^) and GTVnd (<12 cm^3^ or ≥12 cm^3^) with T-, N-category to derive T-N-volume (TNV) prognostic groups in recursive partitioning analysis (RPA) model (Figure 2). The RPA model produced the following four prognostic groups with disparate risks of death: Low risk (N0-2, GTVp <52 cm^3^, GTVnd <12 cm^3^), low–intermediate risk (T1-3, GTVp ≥52 cm^3^, GTVnd <12 cm^3^; or N0-2, GTVp <52 cm^3^, GTVnd ≥12 cm^3^), intermediate risk (N3, GTVp <52 cm^3^, GTVnd <12 cm^3^; or T4, GTVp ≥52 cm^3^, GTVnd <12 cm^3^), and high risk (N0-2, GTVp ≥52 cm^3^, GTVnd ≥12 cm^3^; or N3, GTVnd ≥12 cm^3^). The TNV grouping demonstrated good performance for prognostication (Figure 3); five-year OS rates were 94.6% for low risk, 87.1% for low–intermediate risk, 81.9% for intermediate risk, and 73.6% for high risk, respectively (*p* < 0.001). Clinicopathological characteristics of the TNV risk groupings are shown in Supplementary Table S2.

### 2.4. Performance of TNV Risk Grouping against Eighth Edition TNM Classification and Tumor Volume

**Figure 4:**
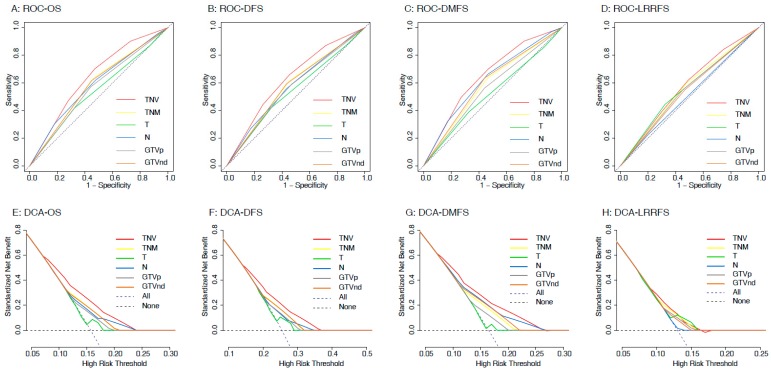
Receiver operating characteristic (ROC) curve and decision curve analyses (DCA) comparing the accuracy of TNV risk stratification system with GTVp/GTVnd and eighth edition TNM stage for predicting overall survival (OS), disease-free survival (DFS), distant metastasis-free survival (DMFS), and local relapse-free survival (LRFS).

Then, we compared the TNV risk stratification against the eighth edition TNM schema and GTVp and GTVnd for prognostication. Receiver operating characteristic (ROC) analyses identified that TNV risk stratification outperformed all other variables for predicting distant metastasis and death (Figure 4A–C), which was confirmed by decision curve analysis (DCA) (Figure 4E–G). The areas under the ROC curve (AUC) are illustrated in Supplementary Table S3; TNV risk stratification had the highest AUC for OS (0.643 vs. 0.541–0.591; *p* < 0.001 for all comparisons), DFS (0.629 vs. 0.545–0.580; *p* < 0.001 for all comparisons), and DMFS (0.652 vs. 0.522–0.621; *p* < 0.001 for all comparisons) among all variables. Notably, although TNV stratification did not outperform TNM schema (AUC: 0.575 vs. 0.560, *p* = 0.201; Supplementary Table S3 and Figure 4D,H), or T category (0.575 vs. 0.561, *p* = 0.376; Supplementary Table S3 and Figure 4D,H) for predicting LRFS, it had better performance than N category, GTVp and GTVnd (0.575 vs. 0.513–0.550, *p* < 0.05 for all comparisons; Supplementary Table S3 and Figure 4D,H). Hence, we concluded that our proposed TNV risk stratification is prone to predicting distant metastasis and death.

### 2.5. Association of Treatment and Survivals in LA-NPC Stratified by TNV Grouping

**Figure 5:**
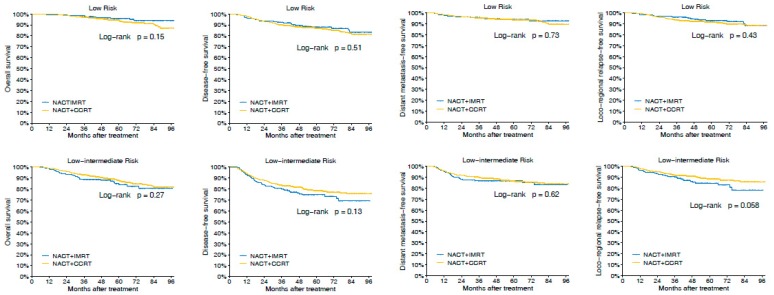

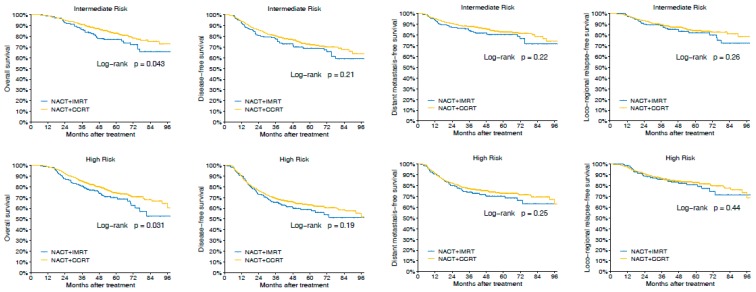
Comparison of the survival rates of neoadjuvant chemotherapy (NACT) + concurrent chemoradiotherapy (CCRT) vs. NACT + intensive-modulated radiotherapy (IMRT) with regard to overall survival, disease-free survival, distant metastasis-free survival, and loco-regional relapse-free survival in patients stratified by TNV groupings.

Next, we examined the interaction between our TNV stratification and efficacy of the different treatment regimes. Overall, we did not observe superiority of NACT + CCRT over NACT + IMRT for OS, DFS, DMFS, and LRFS in the whole cohort (*p* > 0.1 for all, Supplementary Figure S1). Given that LA-NPC is potentially clinically heterogeneous, we then performed subgroup analyses according to the TNV risk stratification to investigate the therapeutic efficacy. For patients with low risk and low–intermediate risk, we still did not observe treatment benefit with NACT + CCRT over NACT + IMRT (*p* > 0.05 for all, Figure 5 and Supplementary Figure S2). Nonetheless, for patients with intermediate risk and high risk, NACT + CCRT was superior to NACT + IMRT in terms of OS (HR 0.68 (95% CI 0.47–0.99) for intermediate risk, 0.73 (0.55–0.97) for high risk; both *p* < 0.05; Figure 5 and Supplementary Figure S2A).

## 3. Discussion

Tumor volume is closely associated with the treatment outcomes of patients with NPC according to previous studies [9,10,11,12,13,14]. In this study, we identified several important findings in a large-scale, real-world database of well-characterized patients. GTVp and GTVnd exerted important prognostic effects on survival and tumor relapse. Additionally, they were independently prognostically significant for OS, DFS, and DMFS, in multivariable analyses. We identified robust thresholds of 52 cm^3^ for GTVp and 12 cm^3^ for GTVnd for risk discretization and applied an intuitive two-tiered classification schema to integrate GTVp, GTVnd, and eighth edition T-, N-categories. Using the RPA method, we constructed a TNV risk stratification that divided the patients into four risk groupings with disparate survival. Additionally, the TNV risk stratification was superior for prognostication (C-index = 0.643) against eighth edition TNM, GTVp, and GTVnd. Importantly, we demonstrated that the TNV stratification was associated with efficacy of chemo-radiotherapy combinations. Based on these data, we can conclude that post-NACT tumor volume is a robust variable to reflect tumor burden and has utility for prognostication. Our TNV risk stratification system is superior to TNM schema and biased towards predicting for occult metastasis/death risk in the era of IMRT. Additionally, the granularity of our TNV groupings caters for a more risk-adapted approach than the current dogma for treatment of LA-NPC.

The prognostic significance of pre-treatment tumor volume is widely accepted and well investigated in previous studies [9,10,11,12,13,14]. Contrarily, the proposition of using post-NACT tumor volume for prognostication in LA-NPC is neglected. Nonetheless, the potential superiority of post-NACT tumor volume as a practical marker for response evaluation and decision-making should not be ignored. Post-NACT tumor volume represents the tumor burden of NPC after NACT, and potentially reflects the response of NPC to NACT. For the patients who remained large tumor volume after NACT, treatment intensification may be proposed since these patients were likely insensitive to chemotherapy. To address these issues, we relied on a large dataset of 4109 cases for which the post-NACT tumor volume at primary site and cervical lymph node were centrally performed. The results of our study suggested that post-NACT tumor volume represented an independent prognostic factor of tumor relapse and death, and the tumor volume demonstrated a similar linear dose (tumor volume)-response relationship for HR_OS_, HR_DFS_, HR_DMFS_, HR_LRFS_. The risks of post-NACT tumor volume for OS, DFS, DMFS, and LRFS increased along with the augment of volume; thresholds of 52 cm^3^ for GTVp and 12 cm^3^ for GTVnd were robust for risk discretization for all treatment outcomes. Taken together, our study represents a critical step toward understanding the prognostic effect of tumor volume on treatment outcomes in LA-NPC after NACT.

The current study also suggests that post-NACT tumor volume has potential utility for decision making in LA-NPC. Currently, clinical trials investigating the role of systemic intensification target the “high-risk” subgroups that are defined by T4N+ and N2-3. [4,5,6,15] Our TNV risk grouping offers a more granular stratification for risk of distant metastasis and death. The low-risk and low–intermediate-risk groups are comprised of patients with N0-2 and GTVp <52 cm^3^ or T1-3 and GTVp ≥52 cm^3^ and GTVnd <12 cm^3^, and combination of NACT and concurrent chemotherapy with IMRT is not superior to NACT with IMRT alone. In contrast, the intermediate-risk and high-risk groups harbor N3 (except for GTVp ≥52 cm^3^) or T4 and GTVp ≥52 cm^3^ and GTVnd <12 cm^3^ or N0-2 and GTVp ≥52 cm^3^ and GTVnd ≥12 cm^3^, and NACT + CCRT is superior to NACT + IMRT alone for improving OS. These findings highlight the utility for adding post-NACT tumor volume in LA-NPC to further inform on treatment intensification and de-intensification.

To our knowledge, this is the first and largest sample-sized study ever reported to evaluate the prognostic effect of the post-neoadjuvant chemotherapy tumor volume in LA-NPC patients. The results of this study are educative and practical. The principal limitations of this study are its retrospective nature; however, this may be weakened due to the large sample size. Thus, the results need validation of further prospective studies. Next, our data are based on a single academic center in an endemic region of China, and thus, external validation with other equally large datasets is needed. Lastly, due to the size of our dataset, it is difficult to determine the level of physician bias in treatment recommendation. Nonetheless, this is not the primary intent of the study, and this will require prospective testing in the setting of a clinical trial.

## 4. Materials and Methods

### 4.1. Patient Selection

We screened 6895 patients with LA-NPC who underwent radical treatment from October 2009 to December 2015 through a NPC-specific database within the big-data intelligence platform at the Sun Yat-Sen University Cancer Centre (SYSUCC). Eligibility criteria included: (1) Biopsy-proven NPC; (2) stages III–IVA disease according to the 8th edition of the International Union Against Cancer (UICC)/American Joint Committee on Cancer (AJCC) TNM staging system [16]; (3) ECOG performance status of 0 or 1; (4) treatment with IMRT; and (5) adequate hematologic, liver, and renal function. Exclusion criteria included: (1) History of previous or synchronous malignant tumors; (2) did not receive NACT; (3) additional use of targeted therapy or immunotherapy; (4) pregnancy or lactation; (5) primary distant metastasis; (6) and insufficient follow-up data. We retrieved data on 4109 eligible patients in the study. This study was approved by the institutional ethics committee and performed in accordance with the guidelines of the World Medical Association (WMA) Declaration of Helsinki (IRB reference No.: YB2018-22). The requirement to obtain informed consent was waived.

### 4.2. Pretreatment and Post Chemotherapy Evaluation

Before treatment, all patients underwent complete pretreatment evaluations, including a complete patient history, physical examination, fiberoptic nasopharyngoscopy, magnetic resonance imaging (MRI) of the nasopharynx and neck, chest, and abdominal computed tomography (CT), emission computed tomography (ECT) or whole-body fluorodeoxyglucose PET/CT, and plasma Epstein–Barr virus (EBV) DNA level. After NACT, MRI of the nasopharynx and neck and fiberoptic nasopharyngoscopy were routinely performed to evaluate the therapeutic effect of chemotherapy.

### 4.3. Tumor Volume Measurement

All patients received radical IMRT and were immobilized in the supine position using a thermoplastic head, neck, and shoulder mask. Contrast-enhanced planning computed tomography (CT; 3 mm-slice thickness) images were obtained from the superior border of frontal sinus to 2 cm below sterno-clavicular joint and transferred to the Monaco treatment planning system (version 3.02; Elekta AB, Stockholm, Sweden).

The post-NACT GTVp and GTVnd were delineated on each slice of planning computed tomography (CT) images, according to the post-NACT MRI image by a radiation oncologist (FPC), and then verified by another radiation oncologist (YS) who specialized in NPC treatment. Enlarged retropharyngeal lymph nodes were encompassed in the GTVp, as it is a difficult issue to clearly distinguish the retropharyngeal nodes from primary tumor in NPC [12,13,14]. For patients whose primary tumor was directly contiguous with the regional nodes, a cut-off level at the mid-C2 vertebra was used to separate the GTVp from the GTVnd, as suggested by Chua et al. [17]. The volumes were calculated by the treatment planning system using the summation-of-area technique, which multiplies the entire areas by the image reconstruction interval of 3 mm.

### 4.4. Radiotherapy and Chemotherapy

Tumor target volumes were delineated according to our institutional treatment protocol, in agreement with International Commission on Radiation Units and Measurements Reports 62 and 83 [18,19]. Two clinical target volumes (CTVs) were delineated according to the tumor invasion pattern [20]. The prescribed doses to the planning target volume (PTV) of GTVp, GTVnd, CTV1 (i.e., high-risk regions), and CTV2 (i.e., low-risk regions and neck nodal regions) were 66–72, 64–70, 60–63, and 54–56 Gy, respectively, in 28–33 fractions. The dose constraints for OARs and PRVs were as described for the RTOG-0225 trial [21]. The targets were treated simultaneously using the simultaneous integrated boost (SIB) technique. All patients were treated following a routine schedule of once daily, over five fractions per week.

Institutional guidelines recommended platinum-based CCRT ± NACT for LA-NPC. Reasons for deviation from the guidelines included individual patient’s refusal, age, or organ dysfunction suggestive of intolerance to treatment. Of the 4109 patients, 3331 (81.1%) received NACT followed by CCRT, and 778 (18.9%) received NACT followed by IMRT. NACT was administered at 3-week intervals for two to four cycles. The regimen of NACT included PF (cisplatin, 80 mg/m^2^ on day 1 with 5-fluorouracil, 800–1000 mg/m^2^ for 96 h of continuous intravenous infusion), TP (cisplatin with docetaxel, 75 mg/m^2^ on day 1), and TPF (cisplatin and docetaxel, 75 mg/m^2^ on day 1, with 5-fluorouracil, 750 mg/m^2^ for 96 h of continuous intravenous infusion). CCRT consisted of platinum every three weeks (80–100 mg/m^2^) for two to three cycles or weekly (30–40 mg/m^2^) for five to six cycles, beginning on first day of IMRT.

### 4.5. Follow-Up

The primary endpoint was OS, which was calculated from start of treatment to the date of death, regardless of cause. Secondary endpoints were DFS, measured from first therapy to evidence of relapse, metastasis, or death; DMFS, to distant metastasis or death; and LRFS, to tumor residue/recurrence or death.

After completing IMRT, patients were followed up at least every 3 months during first 3 years, every 6 months (or until death) for 3 to 5 years, and then annually thereafter. At each appointment, disease status and treatment toxicity were assessed according to our institutional follow-up guidelines. Routine follow-up care included complete head and neck examination, hematology and biochemistry profiles, plasma EBV DNA, chest radiography, ECT, and abdominal sonography. Follow-up nasopharyngeal and/or neck MRI was performed every 6 to 12 months, especially when residual tumor or recurrence were suspected, or RT complications occurred.

### 4.6. Statistical Analysis

The survivals were calculated using Kaplan–Meier analysis, and subgroups were compared using the log-rank test. The clinical features of the different subgroups were compared using χ^2^ analysis and Kruskal–Wallis test. Cox proportional hazards regression was performed to calculate hazard ratios (HR) and 95% confidence interval (CI). Multivariable analyses using cox proportional hazards models were performed to evaluate the independent significance of the potential prognostic factors. The RCS estimated from the full cox regression model were used to evaluate the relationships (in HR) between tumor volume with OS, DFS, DMFS, and LRFS [22]. RPA for OS was conducted to incorporate T-, N-categories with GTVp and GTVnd to derive TNV prognostic group. ROC and DCA were applied to compare the predictive validity of RPA-derived TNV stratification system with GTVp/GTVnd and 8th edition TNM stage. A 2-sided *p* value of <0.05 was considered significant. All analyses were performed using the R 3.4.4 (http://www.r-project.org/) and SPSS 23.0 software (SPSS Inc·, Chicago, IL, USA).

## 5. Conclusions

In conclusion, GTVp and GTVnd represented effective indicators of response evaluation and decision-making in loco-regionally advanced NPC after NACT. We successfully defined optimal tumor volume thresholds and integrated the GTVp and GTVnd with eighth edition TN-categories to construct a two-tiered TNV classification system for LA-NPC. Our TNV risk classification stratifies patients into four TNV risk groupings that are correlated to risks of occult distant metastases and death and outperforms the eighth edition TNM stage for survival prognostication. Additionally, our TNV risk stratification is associated with therapeutic efficacy of treatment regimens; we propose NACT + CCRT for the patients with intermediate- and high-risk groupings, and NACT + IMRT for those with low- and low–intermediate-risk groupings.

## Figures and Tables

**Table 1:** General characteristics.

Characteristic	Patients (*n* = 4109)
Age (years)	
Median	44
IQR	37–52
Sex, *n* (%)	
Male	3071 (74.7)
Female	1038 (25.3)
Family history	
None	3090 (75.2)
Yes	1019 (24.8)
WHO histologic type, *n* (%)	
I	24 (0.6)
II	85 (2.1)
III	4000 (97.3)
T category, *n* (%)	
T1	215 (5.2)
T2	313 (7.6)
T3	2194 (53.4)
T4	1387 (33.8)
N category, *n* (%)	
N0	273 (6.6)
N1	1785 (43.4)
N2	1239 (30.2)
N3	812 (19.8)
Stage, *n* (%)	
III	2091 (50.9)
IV	2018 (49.1)
GTVp (cm^3^), *n* (%)	
Median	48.3
IQR	28.4–77.5
GTVnd (cm^3^), *n* (%)	
Median	11.2
IQR	4.8–22.1
Treatment, *n* (%)	
NACT + CCRT	3331 (81.1)
NACT + IMRT	778 (18.9)

Abbreviation: IQR: Interquartile range; GTVp: primary gross tumor volume; GTVnd: gross tumor volume of lymph node; NACT: Neoadjuvant chemotherapy; CCRT: Concurrent chemoradiotherapy; IMRT: Intensity modulated radiation therapy.

## Supplementary Materials

The following are available online at https://www.mdpi.com/2072-6694/11/11/1632/s1, Supplementary Table S1. Multivariable analyses of T-, N-category and GTVp, GTVnd on clinical outcomes in loco-regionally advanced nasopharyngeal carcinoma, adjusted for age, gender, family history, and treatment scheme. Supplementary Table S2. General characteristics of patients stratified by TNV risk stratification. Supplementary Table S3. Comparison of AUC of TNV risk stratification with TNM categories, GTVp, and GTVnd. Supplementary Figure S1. Comparison of the survival rates of NACT + CCRT vs. NACT + IMRT with regard to (A) overall survival, (B) disease-free survival, (C) distant metastasis-free survival, and (D) loco-regional relapse-free survival in 4109 patients. Supplementary Figure S2. Comparison of benefit of NACT + CCRT vs. NACT + IMRT with regard to (A) overall survival, (B) disease-free survival, (C) distant metastasis-free survival, and (D) loco-regional relapse-free survival in patients stratified by TNV groupings.
