# Supplementary material for: The Role of Post-Neoadjuvant Chemotherapy Tumor Volume for Prognostication and Treatment Guidance in Loco-Regionally Advanced Nasopharyngeal Carcinoma
Source: Cancers (Basel). 2019 Oct 24;11(11):1632. doi: 10.3390/cancers11111632 (PMC6893427; doi:10.3390/cancers11111632)
Supplement: Supplementary file 1 [file cancers-11-01632-s001.pdf]

Supplementary Materials

The Role of Post-Neoadjuvant Chemotherapy Tumor Volume for Prognostication and Treatment Guidance in Loco-Regionally Advanced Nasopharyngeal Carcinoma

Fo-Ping Chen [1,†], Dan-Wan Wen [1,†], Feng Li [1,†], Li Lin [1], Jia Kou [1], Wei-Hong Zheng [1], Li Li [2], Guan-Qun Zhou [1] and Ying Sun [1,*]

Supplementary Tables

Table S1. Multivariable analyses of T-, N-category and GTVp, GTVnd on clinical outcomes in loco-regionally advanced nasopharyngeal carcinoma, adjusted for age, gender, family history and treatment scheme.

	HR (95%CI)	*p*
**OS**		
T stage (T2 vs. T1)	1.17 (0.75–1.83)	0.490
T stage (T3 vs. T1)	1.19 (0.8–1.75)	0.390
T stage (T4 vs. T1)	1.63 (1.07–2.47)	0.022
N stage (N1 vs. N0)	1.09 (0.73–1.63)	0.676
N stage (N2 vs. N0)	1.51 (0.99–2.29)	0.053
N stage (N3 vs. N0)	2.32 (1.51–3.56)	< 0.001
GTVp (≥ 52cc vs. < 52cc)	1.63 (1.35–1.97)	< 0.001
GTVnd (≥ 12cc vs. < 12cc)	1.79 (1.49–2.14)	< 0.001
**DFS**		
T stage (T2 vs. T1)	1.03 (0.72–1.46)	0.890
T stage (T3 vs. T1)	1.13 (0.84–1.52)	0.430
T stage (T4 vs. T1)	1.55 (1.13–2.14)	0.007
N category (N1 vs. N0)	1.15 (0.85–1.57)	0.366
N category (N2 vs. N0)	1.48 (1.07–2.04)	0.018
N category (N3 vs. N0)	1.99 (1.43–2.77)	< 0.001
GTVp (≥ 52cc vs. < 52cc)	1.45 (1.25–1.68)	< 0.001
GTVnd (≥ 12cc vs. < 12cc)	1.7 (1.48–1.96)	< 0.001
**DMFS**		
T stage (T2 vs. T1)	1.06 (0.7–1.6)	0.780
T stage (T3 vs. T1)	1.08 (0.76–1.54)	0.680
T stage (T4 vs. T1)	1.32 (0.9–1.95)	0.156
N category (N1 vs. N0)	1.72 (1.01–2.91)	0.046
N category (N2 vs. N0)	2.77 (1.62–4.74)	< 0.001
N category (N3 vs. N0)	4.01 (2.32–6.91)	< 0.001
GTVp (≥ 52cc vs. < 52cc)	1.52 (1.26–1.84)	< 0.001
GTVnd (≥ 12cc vs. < 12cc)	1.73 (1.44–2.08)	< 0.001
**LRFS**		
T stage (T2 vs. T1)	1.01 (0.58–1.76)	0.970
T stage (T3 vs. T1)	1.1 (0.69–1.75)	0.700
T stage (T4 vs. T1)	1.72 (1.05–2.83)	0.031
N category (N1 vs. N0)	1.07 (0.71–1.61)	0.739
N category (N2 vs. N0)	1.06 (0.69–1.64)	0.782
N category (N3 vs. N0)	1.22 (0.77–1.93)	0.395
GTVp (≥ 52cc vs. < 52cc)	1.23 (0.99–1.52)	0.067
GTVnd (≥ 12cc vs. < 12cc)	1.61 (1.31–1.97)	< 0.001

Abbreviation: GTVp: primary gross tumor volume; GTVnd: cervical lymph node tumor volume; LRFS: loco-regional recurrence-free survival; DFS: disease-free survival; DMFS: distant metastasis-free survival; OS: and overall survival

Table S2. General characteristics of patients stratified by TNV risk stratification.

Characteristic	Low Risk (*n* = 1,004)	Low-Intermediate Risk (*n* = 1,024)	Intermediate Risk (*n* = 825)	High Risk (*n* = 1,256)	*p*
Age (years)					< 0.001
Median	45	43	46	43	
IQR	38–52	37–50	38–53	35–51	
Sex, *n* (%)					< 0.001
Male	697 (69.4%)	779 (76.1%)	610 (73.9%)	985 (78.4%)	
Female	307 (30.6%)	245 (23.9%)	215 (26.1%)	271 (21.6%)	
Family history					0.351
None	751 (74.8%)	754 (73.6%)	637 (77.2%)	948 (75.5%)	
Yes	253 (25.2%)	270 (26.4%)	188 (22.8%)	308 (24.5%)	
WHO histologic type, *n* (%)					0.736
I	8 (0.8%)	4 (0.4%)	5 (0.6%)	7 (0.6%)	
II	22 (2.2%)	26 (2.5%)	14 (1.7%)	23 (1.8%)	
III	974 (97%)	994 (97.1%)	806 (97.7%)	1226 (97.6%)	
T category, *n* (%)					< 0.001
T1	42 (4.2%)	74 (7.2%)	13 (1.6%)	86 (6.8%)	
T2	48 (4.8%)	99 (9.7%)	23 (2.8%)	143 (11.4%)	
T3	736 (73.3%)	798 (77.9%)	63 (7.6%)	597 (47.5%)	
T4	178 (17.7%)	53 (5.2%)	726 (88%)	430 (34.2%)	
N category, *n* (%)					< 0.001
N0	144 (14.3%)	35 (3.4%)	94 (11.4%)	0 (0%)	
N1	574 (57.2%)	479 (46.8%)	451 (54.7%)	281 (22.4%)	
N2	286 (28.5%)	497 (48.5%)	129 (15.6%)	327 (26%)	
N3	0 (0%)	13 (1.3%)	151 (18.3%)	648 (51.6%)	
Stage, *n* (%)					< 0.001
III	826 (82.3%)	958 (93.6%)	0 (0%)	307 (24.4%)	
IV	178 (17.7%)	66 (6.4%)	825 (100%)	949 (75.6%)	
GTVp (cm^3), *n* (%)					< 0.001
<52	1,004 (100%)	677 (66.1%)	111 (13.5%)	417 (33.2%)	
≥52	0 (0%)	347 (33.9%)	714 (86.5%)	839 (66.8%)	
GTVnd (cm^3), *n* (%)					< 0.001
<12	1,004 (100%)	347 (33.9%)	825 (100%)	0 (0%)	
≥12	0 (0%)	677 (66.1%)	0 (0%)	1,256 (100%)	
Treatment, *n* (%)					< 0.001
NACT+IMRT	241 (24%)	206 (20.1%)	148 (17.9%)	183 (14.6%)	
NACT+CCRT	763 (76%)	818 (79.9%)	677 (82.1%)	1,073 (85.4%)	

Abbreviation: LRRFS: loco-regional relapse-free survival; DFS: disease-free survival; DMFS: distant metastasis-free survival; OS: and overall survival; NACT: neoadjuvant chemotherapy; CCRT: concurrent chemoradiotherapy; IMRT: Intensity-Modulated Radiation Therapy; GTVp: gross primary tumor volume; GTVnd: cervical lymph node volume.

Table S3. Comparing of AUC of TNV risk stratification with TNM categories, GTVp, and GTVnd.

	**Overall Survival**			**Disease-Free Survival**			**Distant Metastasis-Free Survival**			**Loco-Regional Relapse-Free Survival**		
	**AUC**	**95% CI**	***p*-Value**	**AUC**	**95% CI**	***p*-Value**	**AUC**	**95% CI**	***p*-Value**	**AUC**	**95% CI**	***p*-Value**
TNV	0.643	0.628 to 0.657	Ref.	0.629	0.614 to 0.644	Ref.	0.652	0.637 to 0.666	Ref.	0.575	0.559 to 0.590	Ref.
TNM	0.586	0.570 to 0.601	< 0.001	0.580	0.565 to 0.595	< 0.001	0.586	0.571 to 0.601	< 0.001	0.560	0.545 to 0.575	0.201
T	0.541	0.526 to 0.557	< 0.001	0.545	0.530 to 0.560	< 0.001	0.522	0.507 to 0.537	< 0.001	0.561	0.545 to 0.576	0.376
N	0.591	0.576 to 0.606	< 0.001	0.576	0.560 to 0.591	< 0.001	0.621	0.606 to 0.635	0.0063	0.513	0.498 to 0.529	< 0.001
GTVp	0.565	0.550 to 0.580	< 0.001	0.564	0.548 to 0.579	< 0.001	0.561	0.545 to 0.576	< 0.001	0.550	0.534 to 0.565	0.032
GTVnd	0.583	0.568 to 0.598	< 0.001	0.578	0.563 to 0.593	< 0.001	0.596	0.581 to 0.612	< 0.001	0.539	0.523 to 0.554	0.001

Abbreviation: AUC: the area under the receiver operating characteristic curve; CI: confidence interval; GTVp: gross tumor volume; GTVnd: cervical lymph node volume.

Supplementary Figures

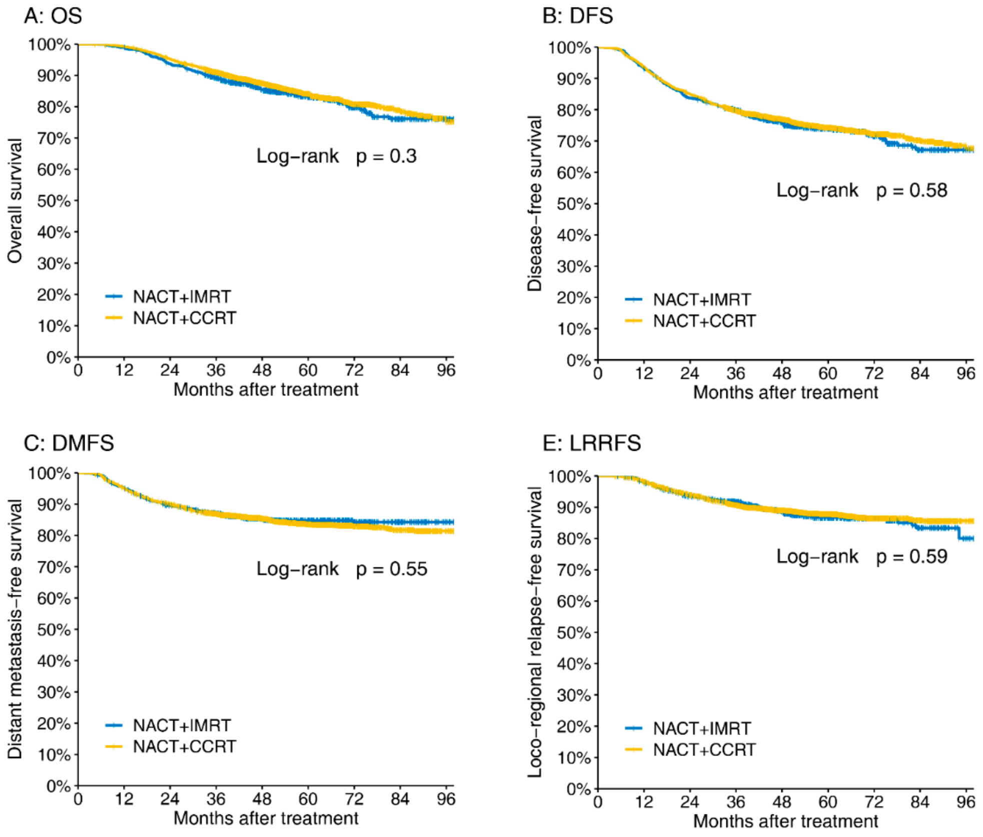

Figure S1. Comparison of the survival rates of NACT + CCRT vs. NACT + IMRT with regard to (**A**) overall survival, (**B**) disease-free survival, (**C**) distant metastasis-free survival, and (**D**) loco-regional relapse-free survival in 4,109 patients.

A Overall survival

Risk group	NACT+IMRT No. of Patients	NACT+IMRT No. of Events	NACT+CCRT No. of Patients	NACT+CCRT No. of Events	HR (95% CI)	*P*
Low Risk	241	12 (5.0%)	763	52 (6.8%)	1.60 (0.84-3.07)	0.157
Low-intermediate Risk	206	30 (14.6%)	818	99 (12.1%)	0.80 (0.53-1.20)	0.247
Intermediate Risk	148	38 (25.7%)	677	113 (16.7%)	0.68 (0.47-0.99)	0.044
High Risk	183	55 (30.1%)	1,073	246 (22.9%)	0.73 (0.55-0.97)	0.031

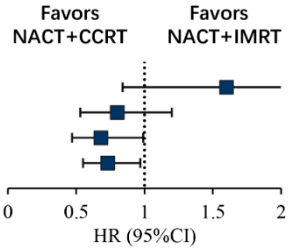

B Disease-free survival

Risk group	NACT+IMRT No. of Patients	NACT+IMRT No. of Events	NACT+CCRT No. of Patients	NACT+CCRT No. of Events	HR (95% CI)	*P*
Low Risk	241	31 (12.9%)	763	107 (14.0%)	1.15 (0.76-1.72)	0.513
Low-intermediate Risk	206	52 (25.2%)	818	167 (20.4%)	0.79 (0.58-1.07)	0.127
Intermediate Risk	148	49 (33.1%)	677	180 (26.6%)	0.81 (0.59-1.12)	0.208
High Risk	183	75 (41.0%)	1,073	385 (35.9%)	0.85 (0.67-1.09)	0.194

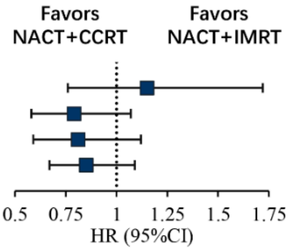

C Distant metastasis-free survival

Risk group	NACT+IMRT No. of Patients	NACT+IMRT No. of Events	NACT+CCRT No. of Patients	NACT+CCRT No. of Events	HR (95% CI)	*P*
Low Risk	241	16 (6.6%)	763	52 (6.8%)	1.11 (0.62-1.97)	0.729
Low-intermediate Risk	206	29 (14.1%)	818	106 (13.0%)	0.90 (0.60-1.36)	0.624
Intermediate Risk	148	31 (20.9%)	677	108 (16.0%)	0.78 (0.52-1.16)	0.22
High Risk	183	55 (30.1%)	1,073	279 (26.0%)	0.84 (0.63-1.13)	0.248

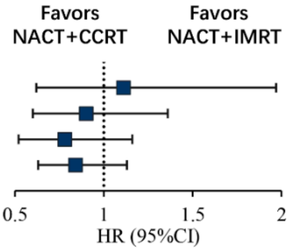

D Loco-regional relapse-free survival

Risk group	NACT+IMRT No. of Patients	NACT+IMRT No. of Events	NACT+CCRT No. of Patients	NACT+CCRT No. of Events	HR (95% CI)	*P*
Low Risk	241	18 (7.5%)	763	68 (8.9%)	1.23 (0.73-2.08)	0.43
Low-intermediate Risk	206	32 (15.5%)	818	86 (10.5%)	0.67 (0.45-1.02)	0.059
Intermediate Risk	148	28 (18.9%)	677	98 (14.5%)	0.78 (0.51-1.20)	0.263
High Risk	183	32 (17.5%)	1,073	172 (16.0%)	0.86 (0.60-1.25)	0.436

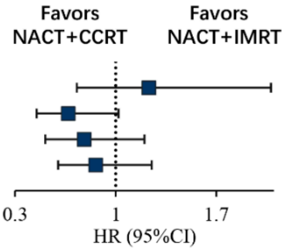

Figure S2. Comparison of benefit of NACT + CCRT vs. NACT + IMRT with regard to (**A**) overall survival, (**B**) disease-free survival, (**C**) distant metastasis-free survival, and (**D**) loco-regional relapse-free survival in patients stratified by TNV groupings.
